# Winter-time solar radiation, precipitation, and psychotropic medication purchases: A cohort study in Finnish public sector employees

**DOI:** 10.1097/EE9.0000000000000369

**Published:** 2025-02-10

**Authors:** Auriba Raza, Timo Partonen, Ville Aalto, Jenni Ervasti, Reija Ruuhela, Magnus Asp, Erik Engström, Jaana Pentti, Jussi Vahtera, Jaana I. Halonen

**Affiliations:** a Department of Psychology, Stress Research Institute, Stockholm University, Stockholm, Sweden; b Department of Healthcare and Social Welfare, Finnish Institute for Health and Welfare, Helsinki, Finland; c Finnish Institute of Occupational Health, Työterveyslaitos, Finland; d Weather and Climate Change Impact Research, Finnish Meteorological Institute, Helsinki, Finland; e Department of Community Planning Services, Swedish Meteorological and Hydrological Institute, Norrköping, Sweden; f Department of Public Health, University of Turku, Turku, Finland; g Centre for Population Health Research, University of Turku and Turku University Hospital Turku, Turku, Finland; h Department of Public Health, Finnish Institute for Health and Welfare, Helsinki, Finland

**Keywords:** Solar radiation, Precipitation, Mental health, Psychotropic medication, Antidepressants

## Abstract

**Background:**

In Northern latitudes, winter is the darkest time of the year, and depressive episodes during winter are prevalent. Although changing weather patterns due to climate change are projected to result in warmer and wetter and, thus, even darker winters, research on the impact of winter-time natural light and precipitation on mental health is scarce. We examined associations of exposure to solar radiation and precipitation with psychotropic medication and antidepressant purchases in winter months.

**Methods:**

Of the 251,268 eligible participants from the Finnish public sector study, aged ≥18 years, 72% were women. Associations for municipality-level 4-week average solar radiation and precipitation with register-based medication purchases from 1999 to 2016 were analyzed using random effects method with Poisson regression. A 6-month washout period with no purchases was applied to each purchase. Confounding by region and year, and effect modifications by sex, age, and socioeconomic status were examined.

**Results:**

No association was observed for an increase in 4-week average of solar radiation by standard deviation (585 kJ/m^2^) with any psychotropic medications (incidence rate ratio: 0.99; 95% confidence interval: 0.98, 1.00) or antidepressants (1.00; 0.99, 1.01). No difference in any psychotropic medication or antidepressant purchases in participants exposed to high solar radiation (≥2000 kJ/m^2^) compared with those with the lowest exposure (<500 kJ/m^2^) was observed. No associations were observed for precipitation.

**Conclusion:**

No evidence linking higher solar radiation exposure to reduced psychotropic medication purchases, nor higher precipitation exposure to increased medication purchases in winter was observed. Further research is needed to validate and expand upon these findings.

**What this study adds**

First to investigate these relationships during winter when lack of light can impact mental health. Our findings did not indicate that higher exposure to solar radiation was linked to a reduced likelihood of purchasing psychotropic medications during the winter months. We addressed the challenges of studying associations between environmental exposures related to climate change and an indicator of severe mental health problems. It contributes to the sparse literature on the impact of meteorological variables on mental health. Environmental Epidemiology, through its global network, can effectively highlight the need for diverse investigations into the relationship between weather conditions and psychiatric disorders.

## Introduction

Seasonal affective disorder (SAD), especially its winter-bound form called winter depression, is significantly prevalent in Northern latitudes.^1–3^ In these regions, winter is the darkest time of the year, with limited daylight hours. This region is experiencing climate change-induced warming at a rate twice as fast as the global average.^4,5^ In the early decades of this century, the ratio of the warming in Finland to global warming relative to 1981–2010 is close to 2 or even higher, but toward the end of the century, the ratio converges toward ∼1.6.^6^ Precipitation is projected to increase and solar radiation to decrease in future winters. While these gradual changes in environmental conditions due to climate change can further exacerbate the symptoms of SAD as well as those of other mood disorders and increase the need for treatment, the research on the impact of climate-sensitive environmental factors on mental health is limited.^7^

The etiology of depressive episodes of depressive or bipolar disorders emerging during the winter comprises numerous hypotheses that suggest the shortage of sunlight and hormonal changes are underlying factors.^8^ Decreased exposure to sunlight decreases serotonin synthesis in the brain, critical in regulating mood, sleep, and appetite.^9,10^ Furthermore, SAD has been directly associated with elevated levels of melatonin,^11,12^ causing fatigue and sleepiness that have also been linked to low exposures to sunlight.^13,14^ While SAD is treated primarily with scheduled exposures to light (i.e., light therapy)^15^ and only secondarily with antidepressants,^16^ research on the associations between the amount of sunlight and psychotropic medication use^17^ is very limited.

Research on the impacts of climatic or meteorological factors on mental health has predominantly focused on the seasonality of mental disorders and their correlation with various meteorological factors in different geographical regions, yielding varying results. A handful of studies have investigated associations of exposure to solar radiation with depression using different exposure windows, resulting in inconsistent findings.^8,18–21^ Moreover, most of the limited literature has relied on self-reported symptoms of depression as an outcome.^8,22–24^ Only two studies, both conducted in Korea, have used hospital registers to define objective outcomes, and they reported conflicting results.^19,21^ To the best of our knowledge, there are no studies investigating the association of precipitation with objectively or subjectively measured mental health.

In this study, we examined associations of winter-time solar radiation and precipitation with the purchase of psychotropic medications, an objective correlate of mental ill-health, using a random effects method in a dataset covering years from 1999 to 2016. We hypothesized that a higher amount of solar radiation and a lower amount of precipitation during winter months would be associated with a lower probability of psychotropic medication purchases.

## Methods

### Study population

**Figure 1.**
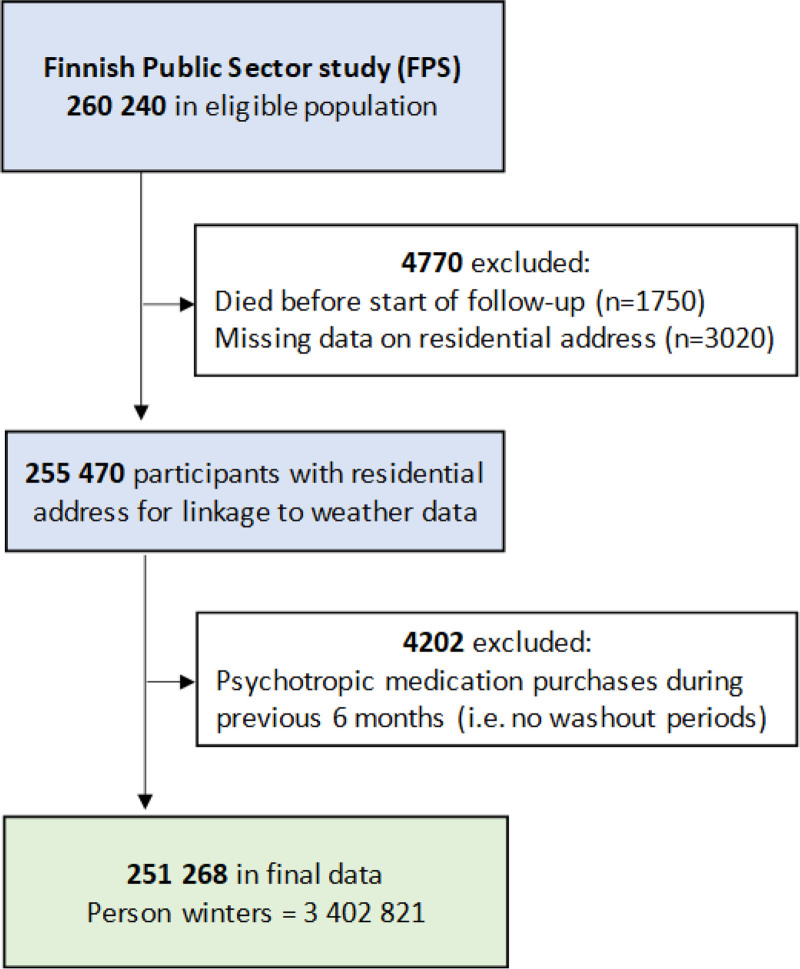
Flow chart of participant selection.

The study population was from the on-going Finnish public sector (FPS) cohort study, which is coordinated by the Finnish Institute of Occupational Health and was initiated in 1997/1998 to monitor the health and well-being of employees in the Finnish municipal sectors, and to examine changes in work characteristics and health risk behaviors over time. Participants are from a diverse range of public sector workplaces such as schools, nurseries, rest homes, health care centers, hospitals, and administration offices. Participants were drawn from employers’ records that have been linked to records from national health and sociodemographic registers. This registered cohort includes 260,240 employees with a job contract between 1990 and 2005. Using personal identification numbers, participants were linked with medication purchases from the Finnish Prescription Register maintained by the Social Insurance Institution of Finland. From this cohort, we selected those aged 18 years or more, with residential address information obtained from the Finnish Population Registry, for winter-time, and at risk of starting a new episode of psychotropic medication (i.e., no purchases within the preceding 6 months). The analysis sample included a total of 251,268 participants from years 1999 to 2016 with 44.2 million weeks of follow-up during 3,402,821 person-winters (1 person-winter equals 13 weeks from the beginning of December to the end of February). Flow chart of participant selection is presented in Figure 1.

The ethical committee of the Helsinki and Uusimaa Hospital District has approved the FPS study (HUS/1210/2016).

### Weather data

Data on global radiation, referred to as solar radiation, and precipitation were obtained from the Finnish Meteorological Institute. Solar radiation data were based on validated 10 km × 10 km gridded daily solar radiation data (kilojoule per square meter [kJ/m^2^]) interpolated from measured values in the Finnish Meteorological Institute observation stations.^25^ Values of the meteorological parameters for each residential municipality were calculated as spatial mean values from the gridded data. Precipitation data were as daily accumulated sums in millimeters (mm). These daily values for both solar radiation and precipitation were used to calculate a 4-week average exposure before each winter-time follow-up week for medication purchases at week-level. For categorical analysis, solar radiation values were categorized as <500, 500–999, 1000–1999, and ≥2000 kJ/m^2^. Precipitation values were categorized as <1.5 (light rain), 1.5–3.5 (medium), and ≥3.5 mm (heavy rain).

### Psychotropic medications

The Finnish Prescription Register,^26^ a pharmacy-claims database, provided data on psychotropic medications. The register contains records of all prescription drug purchases reimbursed to residents in noninstitutional settings. For each drug, the dispensing date and the World Health Organization Anatomical Therapeutic Chemical classification code^27^ are recorded. Any psychotropic medication included N06A, N05A, N05B, and N05C. Antidepressants include N06A. To identify the onset of new treatment episode, we applied a washout period, which we defined as a 6-month period during which there were no purchases of any psychotropic medications. Any subsequent purchase after this period was considered the beginning of a new treatment episode. For each week in the months of December, January, and February in each year, we defined the status of an onset of a new medication episode (yes or no).

### Covariate and effect modifiers

As few personal characteristics can affect municipal-level solar radiation or precipitation, we did not recognize and include any true confounders. However, Finland is a long country extending 1200 km from north to south (60–70^o^N), and the level and seasonal variation in sunlight differs considerably across regions. On the shortest day of the year in December, for example, in the northernmost parts of the country, the length of the day is zero hours, while in the Southern parts, it is nearly 5 hours 50 minutes. Thus, we divided Finland into three regions: northern, central, and southern Finland, and region was used as a covariate. Year was used to adjust for variation within the study period.

Potential effect-modifying factors were identified based on previous literature on outdoor natural light and different mental health endpoints.^3,8,18–20,22,23^ Data on age and sex were from the employers’ personnel registers. Level of education was obtained from Statistics Finland and classified as primary, secondary, and tertiary. Age was classified as <45, 45–54, 55–64, and ≥65 years.

### Statistical analysis

To determine the association of winter-time solar radiation and precipitation with any psychotropic and antidepressant medication purchases separately, we examined the associations of 4-week average exposure with the onset of a new medication episode. Using Poisson regression analyses adjusted for calendar-year and region, we calculated the number of purchases per 1000 person-winters and the corresponding incidence rate ratios (IRRs) and their 95% confidence intervals (CIs). Solar radiation and precipitation were analyzed both as a continuous variable and categorical variable. The estimates for continuous exposures are expressed as IRR per standard deviation increase in 4-week solar radiation and precipitation with 95% CI. For the categorical exposures, the lowest category (solar radiation; <500 kJ/m^2^, rain; <1.5 mm) was used as the reference group. As a point of reference, under clear skies in winter, a typical daily global radiation value in southern Finland in December–January is about 1500 kJ/m^2^.

To examine if the examined associations varied depending on age, sex, and education level, we tested interactions for these variables by computing an interaction term “solar radiation × covariate” and “precipitation × covariate.” For factors with statistically significant interaction (*P* < 0.05), we reported strata-specific effect estimates.

#### Sensitivity analysis

To investigate the influence of residential longevity on the associations, we limited the analysis to those study participants who had lived at the same address for at least 3 years.

Data management and analysis were performed using SAS 9.4.

## Results

### Study population and exposure characteristics

In the analytical sample of 251,268 individuals, the mean age was 43 years, and the majority were women (72%). High percentage of the individuals had high education level and resided in southern Finland (Table 1). A total of 53,099 individuals at least once bought psychotropic medication in the months of December to February between 1999 and 2016. The correlation between solar radiation and precipitation was weak and inverse (−0.25). The mean (standard deviation) 4-week exposure level of solar radiation was 847 (585) kJ/m^2^ and that of precipitation was 1.78 (1.21) mm. There was some variability but no clear trend in the winter-time levels of global radiation and precipitation during the study period (eFigure 1; http://links.lww.com/EE/A327).

**Table 1.** Characteristics of the study participants (N = 251,268)

Variables	Frequency (%)
Sex	
Men	69491 (28)
Women	181777 (72)
Age	
<45	179082 (71)
45–54	44962 (18)
55–64	22558 (9)
≥65	4666 (2)
Level of education	
Tertiary	136612 (54)
Secondary	86491 (34)
Primary	28165 (11)
Region of residence	
Northern Finland	39178 (16)
Central Finland	69186 (27)
Southern Finland	142904 (57)

### Association between winter-time solar radiation and precipitation with psychotropic medication episodes

**Figure 2.**
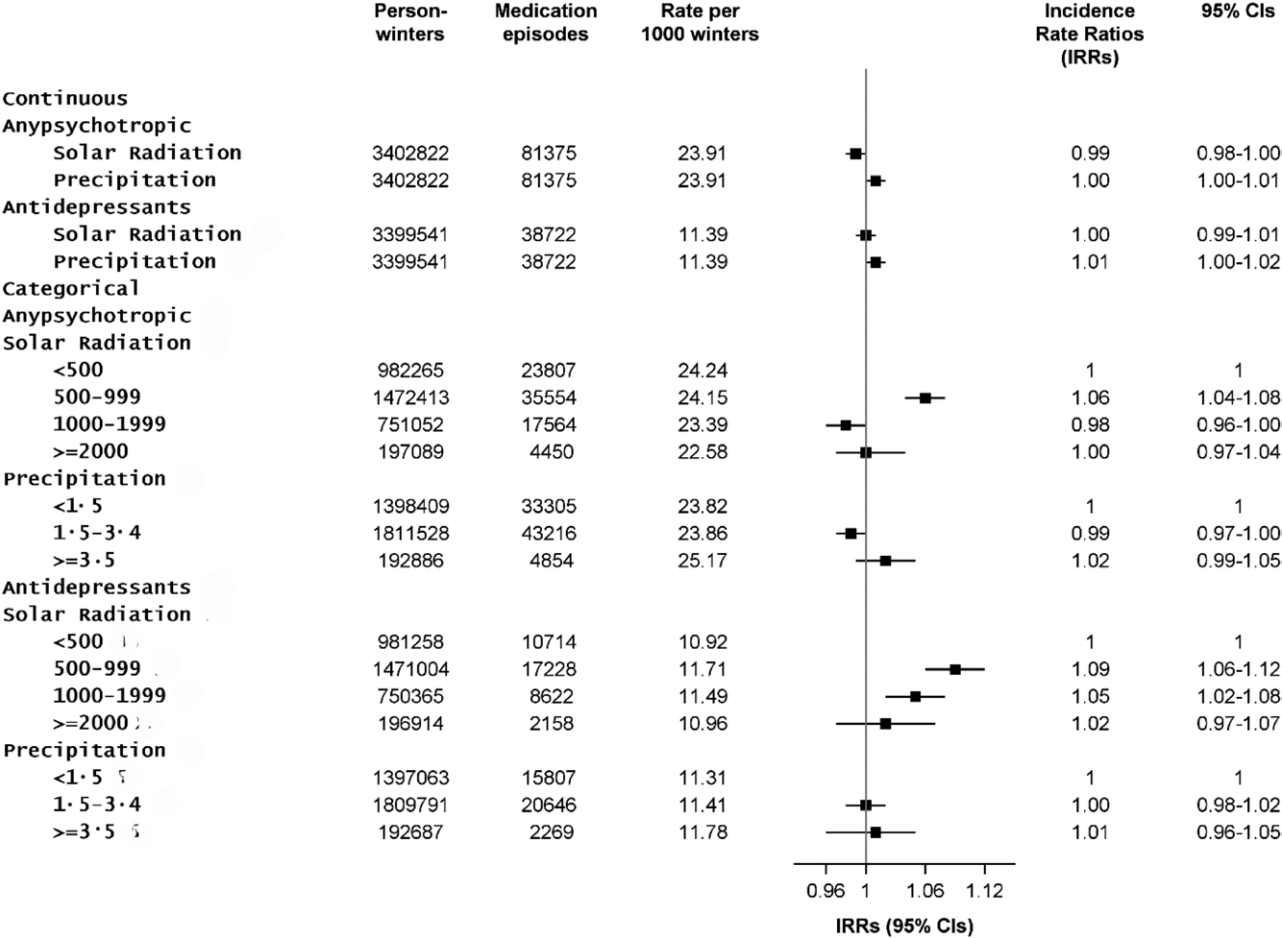
Associations of 4-week solar radiation (kJ/m^2^) and precipitation (mm) with new episodes of psychotropic medications and antidepressants during winter months, per standard deviation increase, adjusted for region and calendar year.

Solar radiation, as indicated by the continuous 4-week average radiation, was not associated with purchases of psychotropic medication (IRR = 0.99; 95% CI: 0.98, 1.00 per SD increase in solar radiation). For precipitation, no association was observed either (Figure 2). In categorical analyses, IRR of psychotropic medication purchases in solar radiation level between 500–999 and 1000–1999 kJ/m^2^, when compared with <500 kJ/m^2^, was 1.06 (1.04–1.08) and 0.98 (0.96–1.00), respectively, but higher radiation levels did not associate with the purchases (Figure 2). For precipitation, there were no associations (Figure 2).

### Association between winter-time solar radiation and precipitation with antidepressant medication episode

We did not observe associations between continuous exposure to 4-week solar radiation or precipitation and antidepressant purchases (Figure 2). In categorical analysis, compared with the lowest solar radiation category, a slightly higher probability of antidepressants purchases was observed for middle-range solar radiation categories but not for the highest category. No difference in association was observed between different categories of precipitation (Figure 2).

### Effect modification

No meaningful differences were observed in the association of solar radiation and psychotropic medication episodes between any subgroups (Table 2). Although we observed statistically significant interaction (*P* = 0.001) with age, one SD increase in solar radiation was associated almost similarly with psychotropic medication purchases among individuals in different age groups (rate ratios in between 0.98 and 0.99). Similarly, no meaningful differences were observed in the association of precipitation and psychotropic medication episodes between any subgroups. All rate ratios were between 0.98 and 1.01 in spite of a statistically significant interaction observed with age and level for education.

**Table 2.** Effect modification by sex, age, and education using continuous variables

	*P*-value for interaction	Incidence rate ratio (95% confidence intervals)
Any psychotropics		
Solar radiation		
Sex	0.184	
Age	0.001	
<45		0.99 (0.98, 1.05)
45–54		0.99 (0.97, 1.00)
55–64		0.98 (0.97, 0.99)
≥65		0.98 (0.96, 1.01)
Education	0.257	
Precipitation		
Sex	0.007	
Male		0.99 (0.98, 1.01)
Female		1.01 (1.00, 1.02)
Age	0.001	
<45		1.01 (1.00, 1.02)
45–54		1.01 (0.99, 1.02)
55–64		0.99 (0.96, 0.99)
≥65		0.98 (0.96, 1.01)
Education	0.003	
Low		0.98 (0.96, 1.00)
Intermediate		1.00 (0.99, 1.02)
High		1.01 (1.00, 1.02)
Antidepressants		
Solar radiation		
Sex	0.190	
Age	0.164	
Education	0.461	
Precipitation		
sex	0.394	
Age	0.07	
Education	0.250	

Effect estimates for the associations between solar radiation and precipitation with psychotropic medication use by subgroups are presented per SD increase in the continuous exposure.

### Sensitivity analysis

The results of the analysis limited to individuals who had resided at the same address for at least 3 years were similar to the main results (eTable 1; http://links.lww.com/EE/A327).

## Discussion

We did not find evidence that higher exposure to solar radiation was associated with a lower likelihood of psychotropic medication purchase during winter months. Stratified analyses by sex, age-group, or education level suggested no obvious differences between the subgroups. Furthermore, no association was observed between precipitation and psychotropic or antidepressive medication purchases.

We hypothesized that higher exposure to solar radiation during winter months would be associated with a lower likelihood of psychotropic medication purchases, and higher precipitation would be associated with a higher likelihood of psychotropic medication purchases, an indicator of doctor-diagnosed mental health problems. We utilized high-resolution individual-level follow-up data for the residential municipality, solar radiation, precipitation, and psychotropic purchases in a between-individual study design. Our findings do not substantiate the hypothesis that solar radiation could be protective for psychotropic medication use during winter months. Most of the existing literature is on the influence of sunlight exposure on symptoms of depression,^8,18–20,24^ while only one prior study has investigated the use of psychotropic medications, namely, self-reported antidepressant usage.^23^ That study reported an association between self-reported time spent outdoors and decreased odds of antidepressant medication use, both in cross-sectional and longitudinal settings throughout a year.^23^ Direct comparison to our findings is challenging, though, since the earlier study used self-reported measures for exposure and outcomes and lacked information on the specific time of the year.^23^

Our observed indication of negative association between higher solar radiation levels and antidepressant purchases may be due to several reasons. One is that we utilized a 4-week exposure period to account for the lag time between initial exposure, the onset of depressive symptoms, seeking medical help, and the subsequent purchase of medication. Estimating how long it takes for a person to seek medical help from a doctor, to receive a diagnosis (e.g., depression) and a prescription, and making the purchase, is a challenge. This timeframe may vary markedly from person to person. In addition, for some study participants, occupational health care was likely provided by private clinics with faster service times, whereas for others, the public health care was only available. Unfortunately, our data did not include information on the availability or use of occupational health care in the study population. Thus, timing of the exposure may have affected our findings suggesting an association opposite to the hypothesis. These challenges might also reflect the current lack of studies on solar radiation and precipitation in the context of psychotropic medication purchases or medication use. Future research should more accurately disentangle the timing of the onset of symptoms to better assess and link the correct exposure data.

Another reason might be that the studied medications might have been used for purposes other than treating SAD. While Finland’s universal drug reimbursement system and the prescription-only nature of psychotropic medications ensure comprehensive data collection, the Prescription Register does not include information on the medical diagnoses associated with these prescriptions. This lack of diagnostic data means we cannot confirm whether the medications were prescribed for SAD, clinical depression, or other conditions that happened to occur during the winter months. Furthermore, individuals with SAD may not seek pharmacological treatment, as light therapy remains the primary treatment for SAD, and some individuals may be unwilling to take medication for this condition. Future research should aim to incorporate diagnostic data to distinguish between different medical conditions and their potential relationships with weather parameters.

Finally, our findings are based on within-winter variability in 30-day average solar radiation and precipitation. While this approach avoids potential confounding by seasonal differences, it may limit the ability to detect larger effect sizes that could arise from broader exposure contrasts, such as those occurring across seasons or over longer time periods. Future research examining these broader contrasts may provide additional insights into the relationship between solar radiation, precipitation, and mental health outcomes.

### Limitations and strengths

This study has some limitations. First, the study cohort primarily consisted of working adults in the female-dominated public sector within the working-age range living mainly in southern or central Finland. Employees may be less vulnerable to developing SAD or other mood disorders due to protective factors such as exposure to outdoor environments and physical activity during commuting, and the benefits of engaging in meaningful work activities. Thus, the findings may not be directly generalizable to the entire population such as unemployed, entrepreneurs, elderly on pension, or children, and those with limited outdoor activity. Second, we used information on residential municipality to link solar radiation and precipitation data with medication purchase that may have led to some exposure misclassification. Furthermore, our analysis did not account for individual-level differences in exposure such as time spent outdoors, or use of adaptive measures (e.g., use of solar lamps), which could influence the true exposure levels. These unmeasured factors could result in differential misclassification that may have either deflated or inflated the estimates. However, controlling for residential longevity had little effect on the observed associations, suggesting that acclimation to local weather conditions is an unlikely source of major bias. Fourth, the results only refer to purchases of medications based on dispensation data, and we could not verify whether and how the dispensed medications were consumed. Fifth, considering that the level of solar radiation varies significantly with latitude, these findings might not be directly generalizable to other populations particularly those living in different latitudes. Finally, it is important to acknowledge that there is an intricate interplay between individual and neighborhood level factors as well as climate, local, and regional weather conditions, which makes the estimation of the association of climatic factors with mental health outcomes challenging.

On the other hand, this study is novel in that, to our knowledge, it is the first study on exposure to solar radiation and precipitation in relation to psychotropic medication purchase during winter months. One strength of the current study was the large study population that was followed over a period of 17 years who contributed to 3,402,822 years of total person winters and 44.2 million follow-up weeks for the outcomes. Second, we used a 6-month washout period to ensure recent severe mental health problems would not affect the examined outcomes and the findings. Third, we tested effect modification by several factors, possibly affecting the examined associations, although unmeasured time-variant and invariant confounding cannot be ruled out.

### Conclusions

With the warming climate, winters in northern European countries are projected to become darker and wetter, which may negatively impact mental health. In this large cohort study from Finland, we did not find evidence to support the hypothesis that higher exposure to solar radiation is associated with a lower likelihood of purchasing psychotropic medications during winter months. As this is the first study of its kind, further research is essential, and we call for additional studies in diverse populations and accurate data to validate and expand upon our findings.

## Conflicts of interest statement

The authors declare that they have no conflicts of interest with regard to the content of this report.

## Supplementary Material


