# Supplementary material for: Winter-time solar radiation, precipitation, and psychotropic medication purchases: A cohort study in Finnish public sector employees
Source: Environ Epidemiol. 2025 Feb 10;9(2):e369. doi: 10.1097/EE9.0000000000000369 (PMC11822337; doi:10.1097/EE9.0000000000000369)
Supplement: Supplementary file 1 [file ee9-9-e369-s001.pdf]

Supplementary material

eTable 1. Associations of four-week solar radiation (kJ/m2) and precipitation (mm) with new episodes of psychotropic medications and antidepressants during winter months, per standard deviation increase, adjusted for region and calendar year. Analysis were limited to those who lived in the same address for at least three years.

		Person-winters	Medication episodes	Rate per 1000 winters	IRR*	95% CI	
**Any psychotropic**							
Solar radiation							
	<500	855355	21251	24.84	1.00		
	500-999	1261682	31301	24.81	1.05	1.03	1.07
	1000-1999	651039	15547	23.88	0.92	0.90	0.94
	>=2000	167880	3883	23.13	1.03	0.99	1.06
Precipitation							
	<1.5	1204132	29377	24.40	1.00		
	1.5-3.4	1562543	38296	24.51	0.99	0.98	1.01
	>=3.5	169281	4309	25.45	1.02	0.99	1.05
**Antidepressants**							
Solar radiation							
	<500	854441	9363	10.96	1.00		
	500-999	1260404	14691	11.66	1.08	1.05	1.11
	1000-1999	650415	7435	11.43	0.97	0.94	0.99
	>=2000	167723	1835	10.94	0.97	0.93	1.03
Precipitation							
	<1.5	1202915	13562	11.27	1.00		
	1.5-3.4	1560967	17803	11.41	1.00	0.98	1.02
	>=3.5	169100	1959	11.58	1.00	0.95	1.04

* Adjusted for calendar year and region.
N(persons) = 250 617
N(winters) = 2 935 956

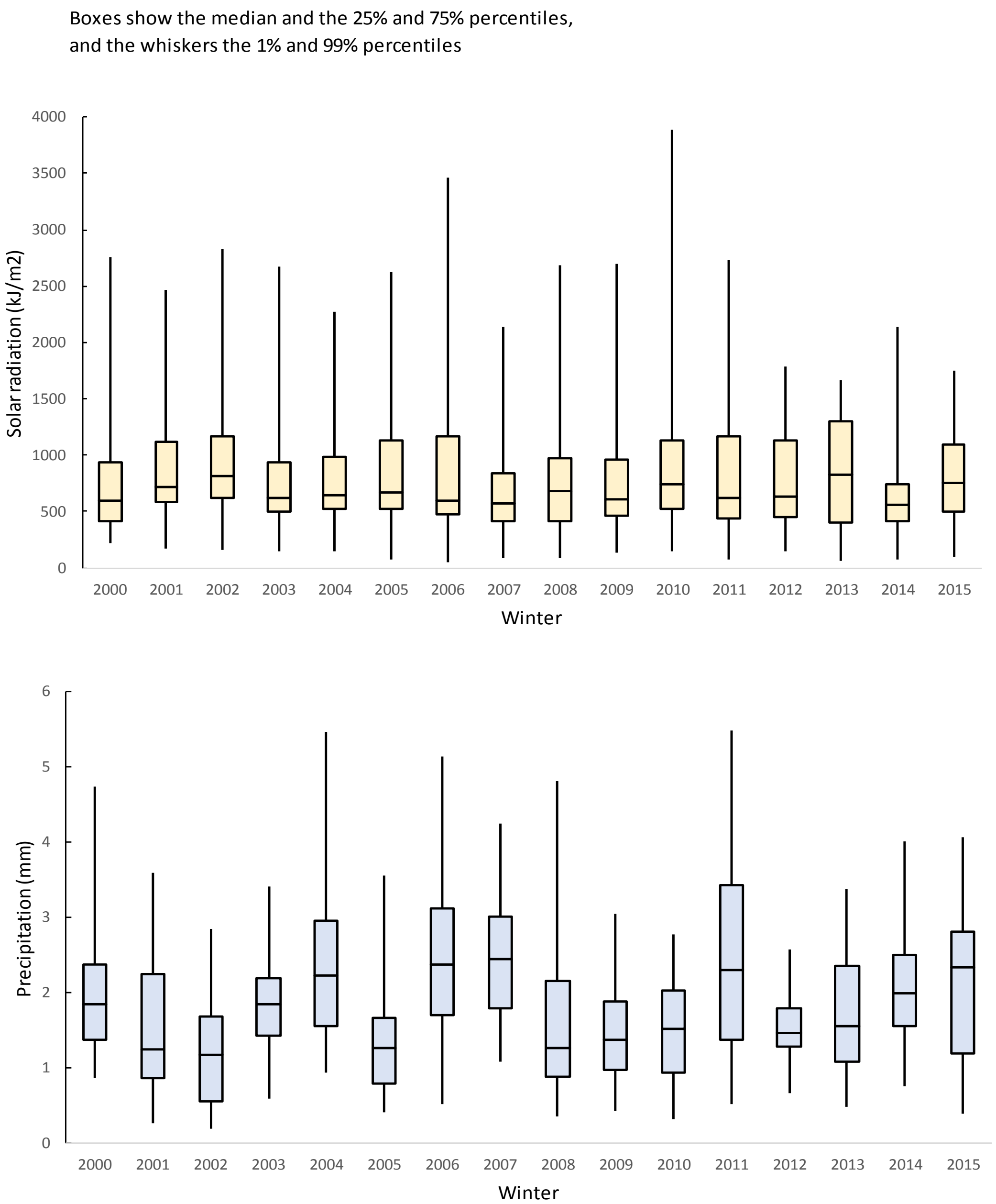

eFigure 1. Annual variation of solar radiation and precipitation between winters from 2000 to 2015
